# Severe Acute Radiation Dermatitis in a Patient with Argyria

**DOI:** 10.1155/2014/154349

**Published:** 2014-03-03

**Authors:** P. Gorayski, M. B. Pinkham, J. B. Muir, A. P. Pullar

**Affiliations:** ^1^Radiation Oncology, Mater Centre, South Brisbane, QLD 4101, Australia; ^2^University of Queensland, St Lucia, QLD 4067, Australia; ^3^Department of Dermatology, Mater Adults Hospital, South Brisbane, QLD 4101, Australia; ^4^Department of Radiation Oncology, Princess Alexandra Hospital, Woolloongabba, QLD 4102, Australia

## Abstract

Argyria is a rare cutaneous condition due to dermal silver deposition leading to irreversible blue-grey discoloration. Acute radiation dermatitis (RD) is an expected toxicity in patients undergoing radiotherapy but it is unknown to what extent argyria affects the onset and severity of RD. We report a patient with argyria treated with concurrent chemoradiotherapy for Merkel cell carcinoma who experienced an unexpectedly brisk and severe RD during treatment. Possible mechanisms for this interaction are considered.

## 1. Introduction

The Therapeutic Goods Administration of Australia has not approved colloidal silver products for therapeutic usage [1]. However, complementary and alternative medicine practitioners may promote colloidal silver tonic for unsubstantiated antineoplastic, antibiotic, or antiviral effects and products labelled as dietary supplements are available in Australia. Chronic use may be associated with neurological, renal, and skin toxicities [2]. Argyria is a condition where irreversible deposition of colloidal silver occurs in the dermis leading to variable blue or slate-grey discoloration. Diagnosis is usually made clinically but silver granules can be seen on skin biopsy as characteristic brown or black granules arranged in single file or clusters surrounding pilosebaceous structures and nerve fibres. The effect of the presence of argyria on the tolerance of the skin to therapeutic radiotherapy (RT) is not known.

Merkel cell carcinoma (MCC) is a rare cutaneous neuroendocrine malignancy with a high propensity for locoregional and distant recurrence. Following wide local excision, adjuvant radiotherapy to the primary site, locoregional nodes, and intervening dermal lymphatics is indicated in most cases [3].

We report on a patient with established generalized argyria who underwent postoperative RT with concurrent chemotherapy for MCC of the head and neck region and developed an unexpectedly brisk and severe skin reaction during treatment. We characterise the reaction and consider whether the presence of argyria may have enhanced his sensitivity to treatment.

## 2. Case Report

A fit 75-year-old man was referred to our institution with a rapidly enlarging violaceous nodule localised to the right temple associated with bleeding but no pain or formication. The patient admitted to using colloidal silver in the preceding ten months for general wellbeing, and clinically there was evidence of generalised argyria. No information was available on dose or formulation because he prepared the solution himself.

He underwent wide local excision of the right temple lesion followed by split skin graft. Histopathology confirmed a 45 mm MCC with focal lymphovascular space invasion. Routine blood tests including haematological, renal, and liver functions were normal. Staging computed tomography of the head, neck, and chest with intravenous contrast and whole body fluorodeoxyglucose positron emission tomography showed no evidence of nodal or distant metastatic disease. Reexcision was required for locally recurrent in-transit disease. No postoperative complications ensued. Further in-transit disease rapidly developed over the right temple and cheek prior to the commencement of RT.

He was ineligible for enrolment into the Trans-Tasman Radiation Oncology Group 09.03 trial [4] due to a previous history of localised prostate cancer but was treated according to this protocol. Adjuvant treatment included RT with concurrent weekly carboplatin (AUC = 2.0) commencing six weeks after surgery. RT was delivered using an intensity modulated simultaneous integrated boost technique delivering 54 Gray (Gy) in 27 daily fractions over 5 and a half weeks to the macroscopic involved sites of disease overlying the right temple and parotid with bolus, 50.4 Gy to the ipsilateral upper neck (nodal groups levels IB-III, Va) and scalp, and 47.6 Gy to the ipsilateral lower neck (nodal groups levels IV-Vb). Three cycles of adjuvant carboplatin (AUC = 4.5) with etoposide (80 mg/m^2^/day over three days) at 3-week intervals were delivered thereafter.

**Figure 1:**
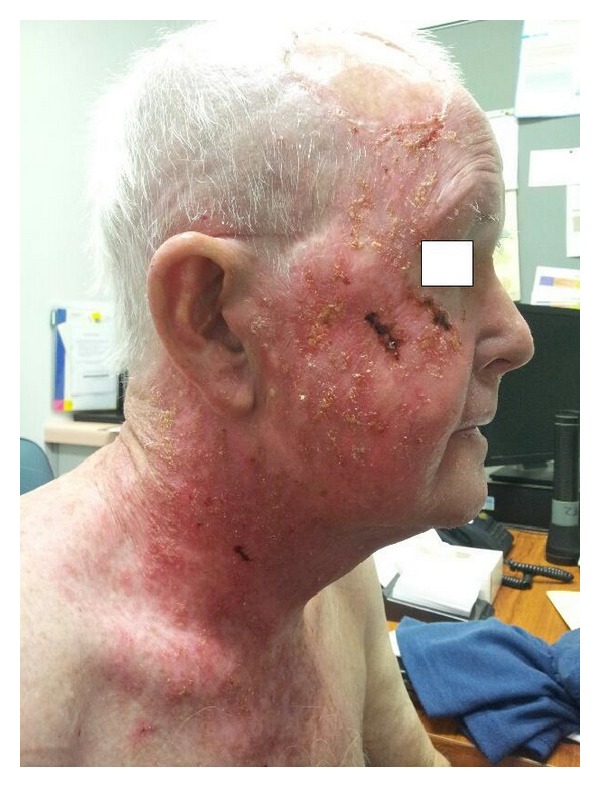
Clinical photograph in week two of radiotherapy showing extensive in-field erythema, areas of dry and confluent wet desquamation, papulopustular changes, and focal regions of haemorrhage.

**Figure 2:**
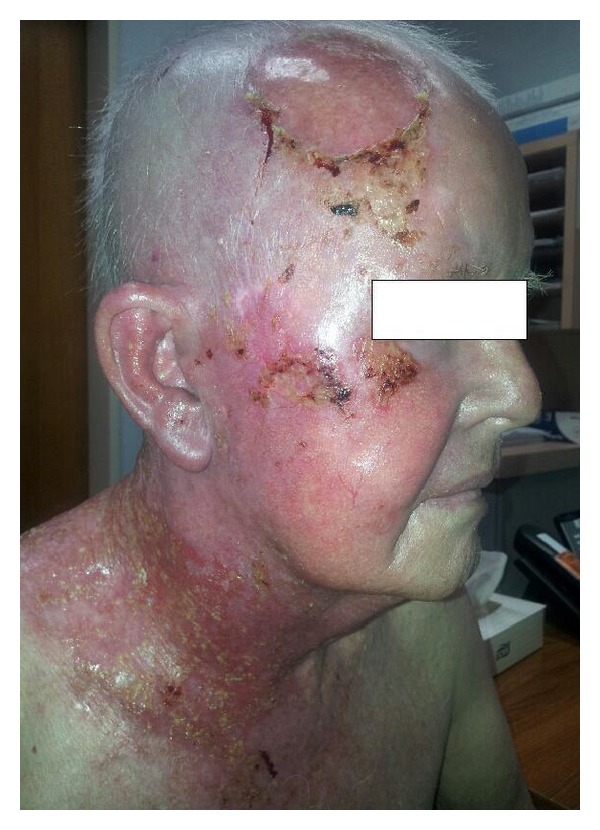
Clinical photograph taken in week five of treatment showing confluent wet desquamation.

**Figure 3:**
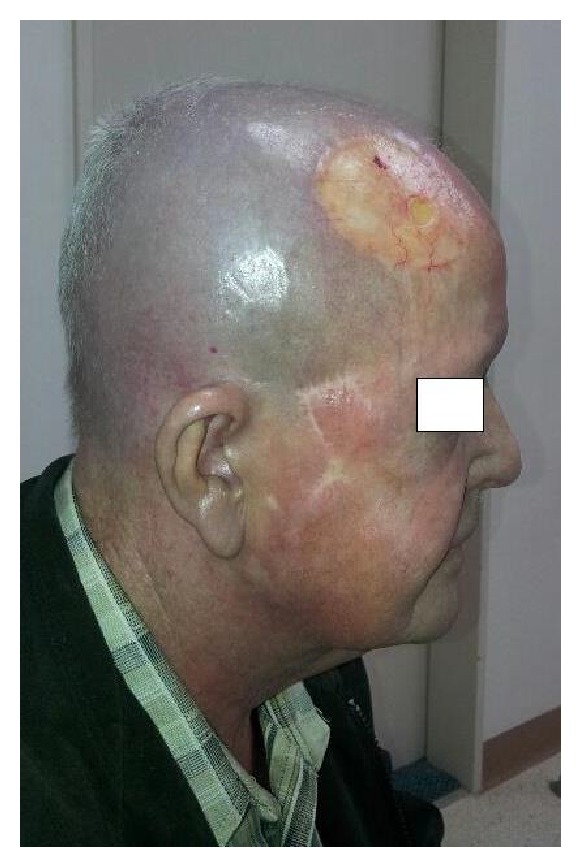
Four months after completing chemoradiotherapy with complete resolution of the radiation dermatitis. Note the small skin ulcer centred in the skin graft.

After one week of RT he developed a brisk papulopustular skin reaction and was commenced on oral empirical cephalexin (Figure 1). In weeks two and three, areas of wet desquamation and spontaneous bleeding were observed, consistent with Radiation Therapy Oncology Group (RTOG) grade four radiation dermatitis (RD) [5]. RTOG grade three confluent ipsilateral oral mucositis was also noted. Treatment parameters were verified and he continued with treatment. By week five, a confluent wet desquamative reaction ensued within the irradiated field (Figure 2), but he was able to complete treatment without interruption. The RD resolved six weeks after RT. Four months after completing treatment, there was no evidence of recurrent disease; however a small asymptomatic skin ulcer developed in the scalp graft (Figure 3).

## 3. Discussion

Acute RD commonly occurs when epidermal keratinocyte loss exceeds the rate at which it is replaced by depleted basal cells. It usually progresses from erythema in weeks one to two, dry desquamation in weeks two to four, and wet desquamation in weeks four to five. The usual time to complete resolution ranges from four to six weeks following completion of treatment. Severity of the reaction depends on a number of factors including skin type, smoking status, medical comorbidities such as diabetes, field size, total radiation dose, overall treatment time, and use of concurrent chemotherapy.

Increased toxicity may be expected when radiosensitizing chemotherapy is given concurrently. In a pilot study of weekly carboplatin with RT for stages one and two of MCC involving eighteen patients, rates of RTOG grade three reactions were limited and no grade four reactions were observed [6]. The skin reaction in this case however was unusually brisk and severe (RTOG grade 4) and occurred after only two weeks of treatment.

Due to the variety of silver-containing products available in Australia and the marked differences in concentrations contained in home-made preparations, we could not quantify silver intake in this patient. Regardless, it was enough to result in the characteristic skin discolouration associated with argyria. In the absence of evidence of a connective tissue disorder [7] or dermatosis to explain the reaction, it is conceivable that the presence of silver in the treated tissues enhanced the RT effect. There are preclinical data of the radiosensitizing effects of silver nanoparticles on glioma cells in vitro [8] and in murine models [9].

We acknowledge that an infective component may have contributed to the severity of the skin reaction in this patient. However, due to the contemporaneous RTOG grade 3 oral mucositis and the use of empiric antibiotics, the skin reaction was more likely secondary to radiation induced changes rather than an infective process alone.

## 4. Conclusion

Chronic colloidal silver may be associated with irreversible bluish discoloration of the skin. In our case, an early brisk and severe papulopustular RTOG grade four RD was observed during RT with concurrent platinum-based chemotherapy. In the absence of an identifiable cause for enhanced radiosensitivity or RD, we postulate that the presence of dermal heavy metal silver deposition potentiated the effects of RT. Patients with argyria should be warned that their skin reaction to RT may be more severe than usual. More studies are needed on the potential radiosensitizing effect of silver which may be exploitable for therapeutic gain.
